# Supplementary material for: Highly Efficient Antibacterial Polymer Composites Based on Hydrophobic Riboflavin Carbon Polymerized Dots
Source: Nanomaterials (Basel). 2022 Nov 18;12(22):4070. doi: 10.3390/nano12224070 (PMC9699046; doi:10.3390/nano12224070)
Supplement: Supplementary file 1 [file nanomaterials-12-04070-s001.zip › nanomaterials-2042999-supplementary.pdf]

Supporting information

Highly Efficient Antibacterial Polymer Composites Based on Hydrophobic Riboflavin Carbon Polymerized Dots

Zoran M. Marković [1,*], Mária Kováčová [2,3], Sanja R. Jeremić [4], Štefan Nagy [5], Dušan D. Milivojević [1], Pavel Kubat [6], Angela Kleinová [2], Milica D. Budimir [1], Marija M. Mojsin [4], Milena J. Stevanović [4,7,8], Adriana Annušová [9,10], Zdeno Špitalský [2] and Biljana M. Todorović Marković [1,*]

1 Vinča Institute of Nuclear Sciences—National Institute of the Republic of Serbia, University of Belgrade, 11000 Belgrade, Serbia
2 Polymer Institute, Slovak Academy of Sciences, Dúbravská Cestá 9, 84541 Bratislava, Slovakia
3 Department of Physical Electronics, Faculty of Science, Masaryk University, Kotlářská 2, 611 37 Brno, Czech Republic
4 Institute of Molecular Genetics and Genetic Engineering, University of Belgrade, Vojvode Stepe 444a, 11042 Belgrade, Serbia
5 Institute of Materials and Machine Mechanics, Slovak Academy of Sciences, Dúbravská cestá 9/6319, 84513 Bratislava, Slovakia
6 J. Heyrovsky Institute of Physical Chemistry, Academy of Sciences of the Czech Republic, Dolejškova 3, 182 23 Praha, Czech Republic
7 Faculty of Biology, University of Belgrade, Studentski trg 16, 11000 Belgrade, Serbia
8 Serbian Academy of Sciences and Arts, Knez Mihailova 35, 11000 Belgrade, Serbia
9 Department of Multilayers and Nanostructures, Institute of Physics, Slovak Academy of Sciences, Dúbravská Cestá 9, 84541 Bratislava, Slovakia
10 Centre for Advanced Materials Application, Slovak Academy of Sciences, Dúbravská Cesta 9, 845 11 Bratislava, Slovakia
* Correspondence: zoranmarkovic@vin.bg.ac.rs (Z.M.M.); biljatod@vin.bg.ac.rs (B.M.T.M.); Tel.: +381−113408582 (Z.M.M.)

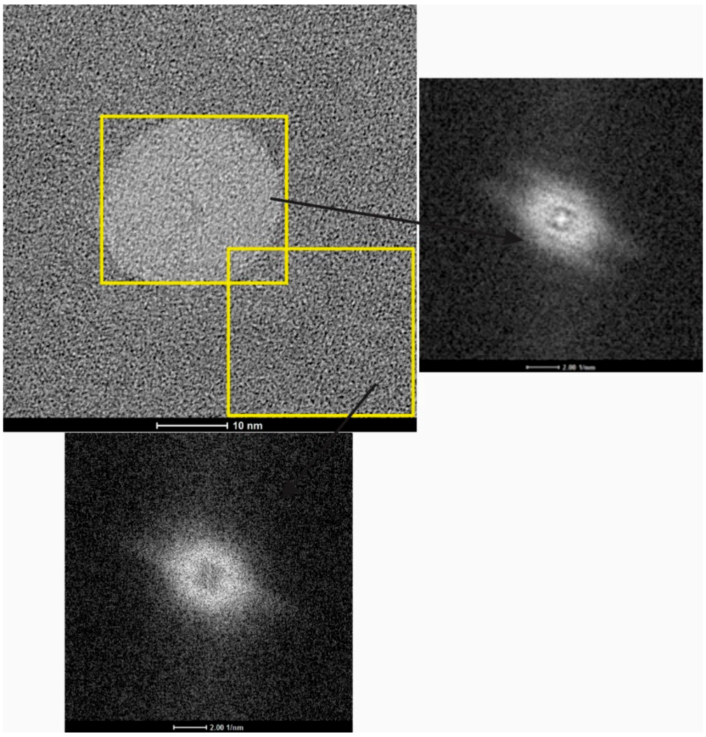

Figure S1. HRTEM micrograph and corresponding electron diffraction patterns of RF-CPDs and TEM supporting grid.

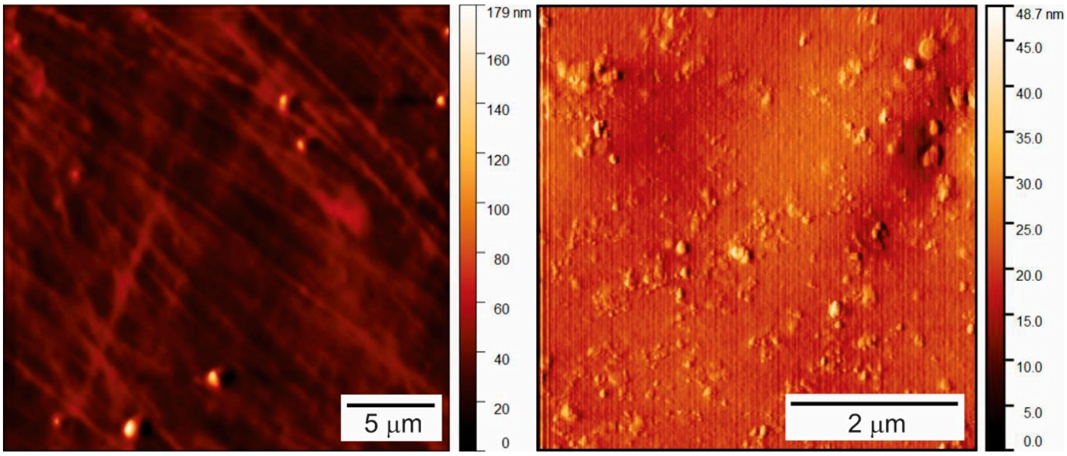

Figure S2. Top view AFM images of a) neat PU and b) RF-CPDs/PU composites.

Table S1. Characteristic bonds detected in the RF-CPDs samples.

Characteristic bonds	Binding energy (eV)	At%
C 1s peak sp^2	284.42	31
C 1s peak sp^3	285.40	48.7
C 1s peak C=O	286.81	20.3
O 1s peak C=O	531.68	100
N 1s peak pyridinic	399.01	45.40
N 1s peak pyrrolic	400.16	48.60
N 1s peak C=N-C	396.72	6.00

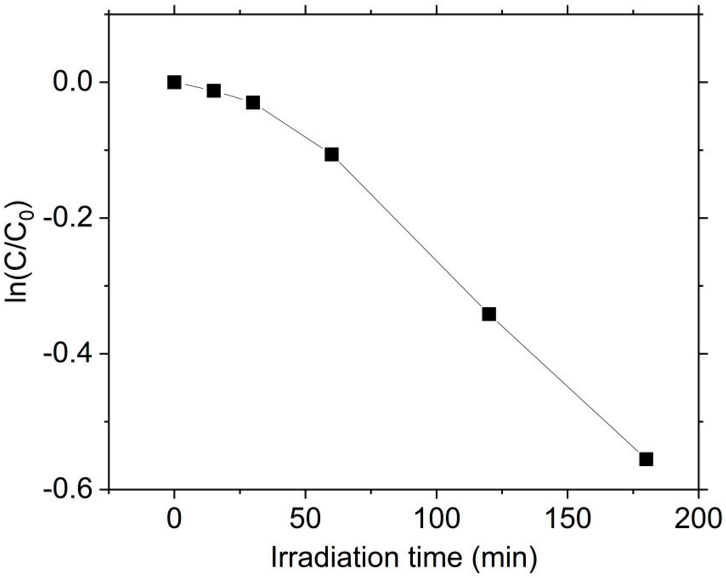

Figure S3. Kinetic rate of RB degradation rate in the presence of RF-CPDs/PU composite under blue light irraditation.

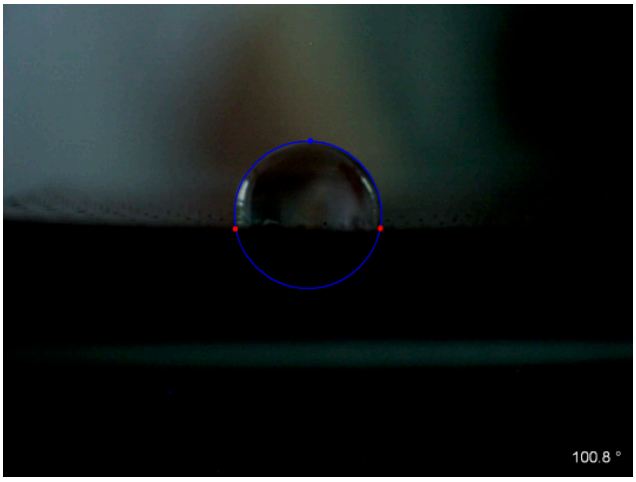

Figure S4. Contact angle measurement of water drop on RF-CPD/PU composites.
